# Fabrication and Assessment of Diosgenin Encapsulated Stearic Acid Solid Lipid Nanoparticles for Its Anticancer and Antidepressant Effects Using *in vitro* and *in vivo* Models

**DOI:** 10.3389/fnins.2021.806713

**Published:** 2022-02-10

**Authors:** Hina Khan, Sadia Nazir, Rai Khalid Farooq, Ishaq N. Khan, Aneela Javed

**Affiliations:** ^1^Atta-ur-Rahman School of Applied Biosciences, National University of Sciences and Technology, Islamabad, Pakistan; ^2^Department of Neuroscience Research, Institute for Research and Medical Consultations (IRMC), Imam Abdulrahman Bin Faisal University, Dammam, Saudi Arabia; ^3^Department of Molecular Biology and Genetics, Institute of Basic Medical Sciences (IBMS), Khyber Medical University, Peshawar, Pakistan; ^4^Healthcare Biotechnology, Atta-ur-Rahman School of Applied Biosciences (ASAB), National University of Science and Technology (NUST), Islamabad, Pakistan

**Keywords:** blood-brain barrier, drug delivery, diosgenin, polysorbate-80, solid lipid nanoparticles, concanavalin-A, sickness mouse model

## Abstract

Inflammatory cascade plays a pivotal role in the onset and progression of major depressive disorder (MDD) and glioblastoma multiforme (GBM). Therefore, questing natural compounds with anti-inflammatory activity such as diosgenin can act as a double-edged sword targeting cancer and cancer-induced inflammation simultaneously. The blood–brain barrier limits the therapeutic efficiency of the drugs against intracranial pathologies including depression and brain cancers. Encapsulating a drug molecule in lipid nanoparticles can overcome this obstacle. The current study has thus investigated the anticancer and antidepressant effect of Tween 80 (P80) coated stearic acid solid lipid nanoparticles (SLNPs) encapsulating the diosgenin. Physio-chemical characterizations of SLNPs were performed to assess their stability, monodispersity, and entrapment efficiency. *In vitro* cytotoxic analysis of naked and drug encapsulated SLNPs on U-87 cell line indicated diosgenin IC_50_ value to be 194.4 μM, while diosgenin encapsulation in nanoparticles slightly decreases the toxicity. Antidepressant effects of encapsulated and non-encapsulated diosgenin were comprehensively evaluated in the concanavalin-A–induced sickness behavior mouse model. Behavior test results indicate that diosgenin and diosgenin encapsulated nanoparticles significantly alleviated anxiety-like and depressive behavior. Diosgenin incorporated SLNPs also improved grooming behavior and social interaction as well as showed normal levels of neutrophils and leukocytes with no toxicity indication. In conclusion, diosgenin and diosgenin encapsulated solid lipid nanoparticles proved successful in decreasing *in vitro* cancer cell proliferation and improving sickness behavioral phenotype and thus merit further exploration.

## Introduction

Depression contributes to poor quality of life in cancer patients, leading to dismal prognosis, increased mortality rate, and risk of suicide (Pinquart and Duberstein, 2010; Linden et al., 2012). Depression risk is two to three times greater in cancer patients when compared with the general public (Brintzenhofe-Szoc et al., 2009; Rooney et al., 2011; Irwin, 2013). Immune dysregulation is proposed as one of the pivotal components of the bidirectional relationship between cancer and depression. Immune system activation in the form of inflammation in the periphery perturbs the balance of neurotransmitters, neurogenesis, and affects neuroplasticity, eventually manifesting into characteristic behavioral deficit (Sperner-Unterweger et al., 2014). On the other hand, inflammation is among seven classical cancer hallmarks, and cancer-induced inflammation can lead to depressive syndromes (Hanahan and Weinberg, 2011). Several signaling pathways have been proposed for the explanation of this bidirectional relationship. Inflammation-induced dysregulation of the kynurenine pathway and particularly changes in IDO1 activity have been documented in cancer and major depressive disorder (MDD) (Raison et al., 2010). Besides, inflammatory cascade and chemotherapeutic regimes also cause or precipitate anxiety and depression in cancer patients. Concomitant therapy with currently available antidepressants has been shown to interact with anticancer chemotherapeutic agents and affect treatment outcomes (Grassi et al., 2018). Moreover, their use in terminally ill patients is also compromised by their slow onset of action and treatment resistance (Block, 2000).

Glioblastoma multiforme (GBM) is a perfect example of this complicated scenario. It is a stage IV astrocytoma and is the most common malignant primary brain tumor, which is uniformly fatal with a mean survival time of 15 months (Koshy et al., 2012; Tamimi and Juweid, 2017). The first-line chemotherapeutic drug for GBM was temozolomide, which contributes toward depletion of adult hippocampal neurogenesis, which remains the pathophysiological basis of depression in GBM patients (Egeland et al., 2017). This situation merits the quest for new interventions that simultaneously target GBM progression and depression or complement each other instead of antagonizing each other. In this regard, therapeutic targeting of inflammatory cascade presents as a promising strategy for the treatment of cancers such as GBM and depression at once. However, the blood–brain barrier (BBB) presents another hurdle to any pharmacological agents to treat pathologies inside the brain. Therefore, anti-inflammatory interventions must be tried in conjunction with improvement in permeability across the BBB (Haar et al., 2012; Tan et al., 2018).

Considering BBB, pharmacological strategies have evolved that are focusing on designing molecules at nano-scale (Harder et al., 2018) to achieve enhanced solubility, target specific controlled release of drug (Parveen et al., 2012; Kumar et al., 2018), and achieve high specificity and receptor-binding affinity (Wrighton et al., 1996) to cross the BBB. In this regard, various nanocarriers have been formulated and in use such as liposomes, polymer nanoparticles, and solid lipid nanoparticles. However, among lipid nanoparticles, solid lipid nanoparticles (SLNPs) have shown better efficacy owing to their ability (Müller et al., 2000; Kumar et al., 2020) to cross the BBB due to their lipophilic nature and entrapment efficiency for both hydrophilic and hydrophobic substances (Bodor and Buchwald, 1999). SLNP has overcome the limitations that other nanoparticles exhibited by having the following properties: greater stability, high biocompatibility, low toxicity, high drug loading capacity, controlled release of drug, compatibility with most drugs, and stable shelf life (Mehnert and Mäder, 2001; Kumar and Randhawa, 2013). As compared with other nanoparticles, SLNPs are formulated from FDA-approved low-cost safe excipients and can be formulated by various yet simple protocols that mainly focus on high drug load, biocompatibility, and stability (Kumar et al., 2020). Excipients like polysorbate-80 (P80) coating can facilitate drugs to cross the BBB efficiently (Alyautdin et al., 1997). Owing to its unique characteristic to emulate the nano-carrier as low-density lipoprotein (LDL), it is recognized by the LDL receptors of the BBB as its own ligand and taken up via endocytosis (Gao and Jiang, 2006; Jones and Shusta, 2007).

Diosgenin, a natural steroidal saponin, possesses anti-inflammatory properties and has been used in traditional medicines for its anti-diabetic, anti-hyperglycemic, anti-hypercholesterolemic, and anti-oxidative properties (Raju and Mehta, 2009; Jesus et al., 2016). In addition, the anticancer activity of diosgenin against breast carcinoma, gastric cancer, colon cancer, prostate cancer, squamous cell carcinoma, and hepatocellular carcinoma has also been documented (Cai et al., 2020). However, its anticancer potential against GBM and inflammogen-induced depression has not been well documented. Therefore, considering inflammation as a pivotal component of disease pathogenesis in GBM and depression, limitations of currently available therapeutic interventions, and anti-inflammatory potential of diosgenin, the current study aimed to evaluate the therapeutic potential of diosgenin against GBM and depression.

In this regard, we examined the cytotoxic potential of diosgenin against GBM (*in vitro*) using U87-MG cell line and antidepressant potential (*in vivo*) using the sickness behavior model in Balb/c mice. In addition, to enhance its permeability across the BBB, diosgenin entrapped solid lipid nanoparticles were prepared and evaluated for anticancer and antidepressant potential.

## Materials and Methods

Lecithin and reagent-grade stearic acid (95%) were from Sigma-Aldrich; polysorbate-80 was from Sigma-Aldrich; isopropanol, phosphate-buffered saline (PBS), HPLC-grade acetonitrile, and diosgenin (93% purity) were from Sigma; formaldehyde solution (37%) was from Riedel-de Haen; extra pure chloroform was from Riedel-de Haen; paraformaldehyde was from Daejung; DMEM (1 ×) + GlutaMAX (1 g/L D-glucose, 110 mg/L sodium pyruvate) was from Gibco; 10% fetal bovine serum was from Gibco; Penstrep was from Gibco; and 0.4% trypan blue was from Gibco.

### Preparation of Stearic Acid Solid Lipid Nanoparticles

**FIGURE 1:**
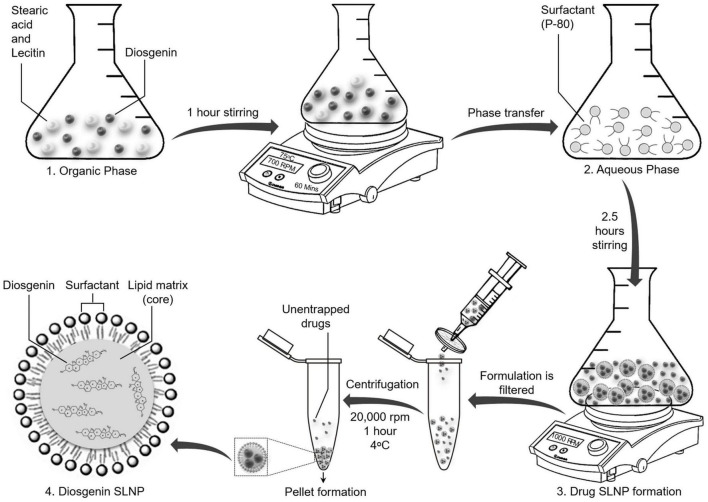
Schematic diagram showing synthesis of solid lipid nanoparticles with encapsulated diosgenin. Lipids and diosgenin in organic phase were added in isopropanol. Hot organic phase was injected in aqueous phase with surfactant Tween 80 for coating purpose. Diosgenin solid lipid nanoparticles were formed following evaporation, centrifugation, and drying of pellet. Final structure of diosgenin solid lipid nanoparticles with Tween 80 coating has been presented at stage 4.

SLNPs encapsulating diosgenin were prepared as shown in Figure 1, using reported protocols of solvent emulsification/evaporation method (Kushwaha et al., 2013; Pooja et al., 2016) with few modifications. An organic solution containing stearic acid (380 mg), lecithin (200 mg), and diosgenin (300 mg) in isopropanol (20 ml) was stirred on a hot plate magnetic stirrer at 700 rpm and 75°C for 60 min. The organic phase (6 ml) was injected into an aqueous solution of 1 g of P80 emulsified in phosphate-buffered saline (SALINE) and was stirred for 2.5 h at 1,000 rpm while keeping the solution temperature at 75°C. Double-distilled cold water (15 ml) was added to the final mixture and was passed through a 0.2-μm syringe filter, centrifuged at 20,000 rpm for 1 h at 4°C, and dried to obtain a pellet of SLNPs. Diosgenin-free SLNPs were also formulated by the same procedure.

### Characterization of Diosgenin Encapsulated Stearic Acid Solid Lipid Nanoparticles

#### Scanning Electron Microscopy

The surface morphology and size were analyzed through Scanning Electron Microscopy (SEM). One milligram of blank SLNP and diosgenin incorporated SLNP were suspended in 2 ml of saline solution. The solution was kept in ice and sonicated well to get an evenly distributed SLNP suspension. A drop of solution was placed on a square-cut silicone slide, which was fixed on a stub with double-sided adhesive carbon tape. With an ion sputter, the samples were coated with gold particles under vacuum before SEM analysis. Samples were observed at different resolutions, and photographs were taken at various magnifications with spatial resolution ranging from 2 to 0.5 μm (Lobovkina et al., 2011; Shazly et al., 2018).

#### Fourier-Transform Infrared Spectroscopy

Fourier-Transform Infrared (FTIR) spectroscopy was performed using a Nicolet Magna-IR 560 optical bench (Madison, WI, United States), where the FTIR spectra were recorded to determine the coupling between lipid particles and diosgenin through specific peaks. SLNP spectra were recorded by the KBr disc method prepared by pressing KBr at 5 kpsi hydraulic pressure. A droplet of the sample was placed on the disc and was analyzed at wave number 4,000–400 cm^–^ (Son et al., 2007).

#### X-Ray Diffraction Analysis

X-Ray Diffraction (XRD) analysis of SLNP was performed to study the crystalline form of diosgenin dispersion in SLNP. This was performed by placing a 0.5-mm-thick sample disc in the disc holder. The samples were exposed to a monochromatic, collimated X-ray beam emitted from a Rigaku-Denki RU3 rotating copper anode generator and brought to a line focus by a diffraction camera containing a single vertical Franks’ mirror to enhance the monochromaticity of the beam. Diffraction patterns were recorded for approximately 1 h with diffraction angles ranging from 5 to 65° with a one-dimensional quartz wire detector. The graphs were attained with MiniFlex software (Kushwaha et al., 2013).

#### Zeta Potential and Zeta Size Measurement

Zeta potential of SLNPs were performed to determine its stable nature and charge present on its surface. Zeta size was determined to get the average size of the SLNPs. These were analyzed by a Nano-ZS zeta sizer (Malvern Instruments, Malvern, United Kingdom) at 25°C. The SLNPs were suspended in a saline solution before analysis, and graphs were obtained for their size and potential charge (Li N. et al., 2009). Values of samples were taken as the mean SD of three replicates. Through the polydispersity index, the width of the total size distribution was measured.

### Evaluation of Diosgenin Release and Entrapment Efficiency

Diosgenin release efficiency from SLNP was determined for 1, 5, and 10 mg of diosgenin incorporated SLNP. The drug-loaded SLNPs were dissolved in 1 ml acetonitrile and were kept at room temperature for an hour. Then SLNPs were centrifuged for 30 s at 1,500 rpm, and the supernatant was collected. Twenty microliters of the supernatant was injected to HPLC, and separation was carried out on the C18 column with flow rate 3 min/1 ml, 210 nm UV range, and retention time of 6.01 min. All the aforementioned measurements were done in triplicates. The efficiency of released diosgenin from SLNP was calculated by Beer’s Law concept.


y=mx+c

For entrapment efficiency, SLNP suspension was centrifuged for 30 min at 15,000 rpm. The supernatant was passed through a 0.2-μm syringe filter. Then 100 μL of the supernatant was distributed in 900 μL of acetonitrile, and unentrapped drug absorbance was observed through HPLC at 210 nm with above-set parameters (Dora et al., 2010; Arora, 2012). The entrapment efficiency of SLNP was calculated as follows:


$$\mathrm{Entrapment}\mathrm{efficiency}\left(\%\right)=\frac{\begin{array}{c} \mathrm{The}\,\mathrm{total}\,\mathrm{amount}\,\mathrm{of}\,\mathrm{drug}-\mathrm{Total}\,\mathrm{amount}\,\mathrm{of}\\ \mathrm{unentrapped}\,\mathrm{drug}\,\mathrm{in}\,\mathrm{suspension} \end{array}}{\begin{array}{c} \mathrm{Total}\,\mathrm{amount}\,\mathrm{of}\,\mathrm{drug} \end{array}}\times 100$$

### Percent Yield of Diosgenin Stearic Acid Solid Lipid Nanoparticle

The yield of diosgenin SLNPs were calculated by weighing the dried-up diosgenin incorporated SLNP that was recovered through centrifugation and dividing it with the total amount of drug and lipids added initially.


$$\mathrm{Percentage}\,\text{yield}=\frac{\mathrm{Weight}\,\mathrm{of}\,\mathrm{dried}\,\mathrm{drug}\,\text{SLNPs}}{\mathrm{Total}\,\mathrm{weight}\,\mathrm{of}\,\text{drug}+\text{lipids}}\times 100$$

### *In vitro* Cytotoxicity Assay

MTT, which assessed diosgenin cytotoxicity (3-[4,5-dimethylthiazol-2-yl]-2,5-diphenyl tetrazolium bromide), is a colorimetric assay on human primary glioblastoma cell line (U87 MG). A quantity of 1 × 10^4^ viable cells/well was plated into a 96 well-plate in 100 μL of cell culture media and incubated overnight for attachment into which different concentrations (ranging from 0.0002 to 2 mg/ml) of diosgenin, blank SLNP, and diosgenin incorporated SLNPs were added. Saline was used as a control, and doxorubicin was used as a positive control. After incubation of cells for 24 h in a 5% CO_2_ incubator, 15 μL of MTT (5 mg/ml) reagent was added into each well. The plates were again incubated for 3 h with the same parameters. When purple precipitates (formazan) were visible, all the solution from plates were aspirated, and the formazan crystals were dissolved in 100 μL of DMSO while kept in the dark at room temperature for an hour. Cell viability was assessed through the absorbance rate of formazan in the well, which was recorded at 550 nm in a microplate reader (Bahuguna et al., 2017). Data were compared and presented as percent relative viability for drug treatment. Percentage cytotoxicity was calculated as


$$\%\mathrm{Cell}\text{viability}=\frac{\left(\text{As}-\text{Ab}\right)}{\left(\text{Ac}-\text{Ab}\right)}\times 100$$

where As, Ab, and Ac correspond to the absorbance value of the sample, blank, and control, respectively.

The IC_50_ was extrapolated from the dose–response graph. The values were calculated using regression analysis of the PRISM program.

### *In vivo* Analysis: Ethics Statement and Animal Housing

A total of 80 6-week-old male Balb/c mice were used for this study, which were provided by the National Institute of Health, Pakistan. Their weight ranged from 25 to 35 g. Animals were housed in polycarbonate cages (475 × 350 × 200 mm) in groups of five. They were kept under standard conditions with a light–dark schedule maintained at 12 h, room temperature maintained at 25 ± 2°C, with access to feed and water *ad libitum*. All experimentations were performed according to the protocols that were approved by the Internal Review Board (IRB), National University of Sciences and Technology (NUST), Pakistan, and it was conducted under the rules and regulations of NIH, United States (National Research Council (US) Committee for the Update of the Guide for the Care and Use of Laboratory Animals, 2011).

### Animal Grouping and Model Development

**FIGURE 2:**
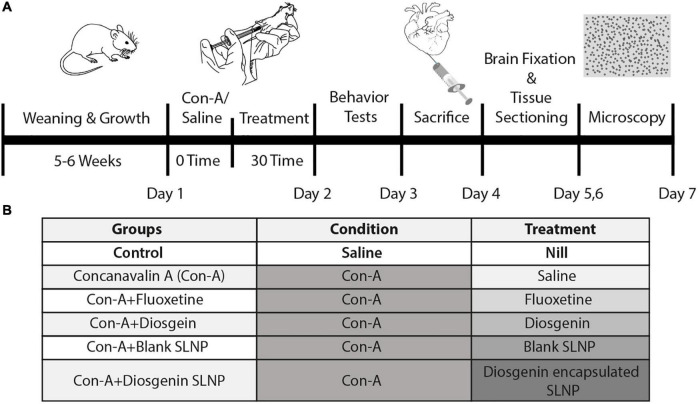
**(A)** Seven-day schematic schedule of BALB/c mice model of sickness behavior and treatment. Five groups of 8-week-old BALB/c mice were exposed to Con-A (10 mg/kg body weight) to induce sickness behavior followed by subsequent pharmacological treatment. Behavior tests were performed on day 2 followed by histopathological analysis of the brain sections. **(B)** Grouping of mice was done on the basis of pharmacological treatment. Control group was given IP injection of saline (10 ml/kg body weight). The rest of the groups were injected with Con-A and after 30 min were treated with respective pharmacological treatments of fluoxetine, diosgenin, blank SLNP, and diosgenin SLNP (10 ml/kg body weight).

Mice were divided into six groups, as illustrated in Figure 2, control (saline), Con-A, Con-A + fluoxetine, Con-A + diosgenin, Con-A + blank SLNP, and Con-A + diosgenin SLNP. Except for the control group, the rest of the five groups were given intraperitoneal (IP) injections of concanavalin-A (Con-A) (10 mg/kg body weight prepared in 0.9% normal saline @ 10 ml/kg body weight). After 30 min of Con-A injections, these groups were intraperitoneally injected with fluoxetine (20 mg/kg body weight prepared in 0.9% normal saline @ 10 ml/kg body weight), diosgenin (100 mg/kg body weight prepared in 0.9% normal saline @ 10 ml/kg body weight), blank SLNP, and diosgenin incorporated SLNP. Table 1 shows the total concentration of drugs dissolved in saline for eight mice in each group.

**TABLE 1:** SLNP efficiency of entrapment, release of diosgenin, and yield of diosgenin SLNP has been quantified with HPLC.

SLNP composition (mg)	Diosgenin entrapment efficiency	Diosgenin release efficiency (10, 5, 1 mg)	Percentage yield	Unentrapped diosgenin (residual)	Unentrapped diosgenin in solvent
*Stearic acid/lecithin/diosgenin* *(200:380:300)*	56 ± 5%	93.7, 97.5, 99.5%	51.70%	25%	19%

### Behavior Tests

All behavior tests were performed in a separate room 24 h after the second injection. Mice were allowed to habituate in the room for 30 min before starting the tests.

#### Open Field Test

Open field test was conducted to quantify anxiety-like behavior in mice as conducted by Gould et al. (2009). The test mouse was placed in the center of the square-shaped 40 × 40 × 40 cm open-field arena, and time spent was recorded for 5 min. Total time spent in the central square was measured.

#### Splash Test

The splash test was conducted to evaluate motivational behavior as described previously with slight modifications (Isingrini et al., 2010). Grooming behavior is a form of motivational behavior and parallel to apathic behavior symptoms of depression. In the test session, 10% sucrose solution was sprayed once on the dorsal coat of the test mouse in the home cage. Evaporation of sucrose solution makes the fur dirty and sticky and instigates the subject for self-grooming. Followed by a spray of sucrose solution, a test session was recorded for 5 min to evaluate motivational behavior by quantifying time spent in self-grooming.

#### Tail Suspension Test

The tail suspension test was conducted as described previously (Steru et al., 1985). This test was used to quantify resignation behavior. The test mouse was suspended at 40 cm height from the ground by its tail on a firm flat wooden support with the help of paper tape. The mobility time was recorded for 6 min of the test session. The mouse was considered immobile when it ceased all motions and hanged passively.

#### Forced Swim Test

Forced swim test was performed to evaluate depressive-like behavior as previously described (Szewczyk et al., 2010). The test mouse was placed in a transparent cylinder (25 cm height and 15 cm diameter) containing 12 cm of water at 24 ± 1°C. The test was conducted for 6 min. After initial movements to escape from the water, mice develop immobility. Mobility time of 6 min in total was measured to evaluate the antidepressant effect of treatment.

#### Social Interaction Test

This test comprised two sessions: social interaction session one and social novelty session two. Each session was recorded for 10 min, and there was a 20-min interval between the two sessions. The apparatus used comprised a three-chamber (19 × 45 cm) box with an open middle section. The test mouse was placed in the central chamber and given 5 min as acclimatization time, followed by session one. Social preference was tested in session between an empty wired cage and a similar cage with a stranger mouse S1. In session two, the social novelty was tested; another stranger mouse (S2) was kept in the empty cage, while a familiar mouse from the session (S1) remained in the same cage as in session one. The interaction time of test mice was recorded for 10 min with empty cage, S1, and S2 (Brenes Sáenz et al., 2006).

### Histological Analysis

#### Perfusion and Brain Tissue Preparation

Brain samples were collected by following the perfusion protocol as described by Gage et al. (2012). Mice were anesthetized by injecting (IP) a cocktail of ketamine + xylazine (87.5 + 12.5 mg/kg). Mice were transcardially perfused (left ventricle) with 80–90 ml of cold saline solution (0.9% NaCl solution) and 90 ml of 4% paraformaldehyde solution (PFA). Brains were harvested and kept in 4% PFA solution for 24 h at 4°C, which was followed by dehydration with different concentrations of ethanol and finally with xylene for 10 min.

#### 70% Ethanol > 80% Ethanol > 85% Ethanol > 90% Ethanol > 100% Ethanol

Samples were fixed in paraffin wax and sliced on SLEE Mainz (CUT6062) microtome, where each slice was 3 μm thick. The slices were deparaffinized on a hot plate at 60°C and rehydrated in xylene and ethanol. The slices were then exposed to H&E staining to stain the tissues. Once they were stained, slides were observed under a microscope at different magnifications.

### Evaluation of Diosgenin Concentration in Blood and Organs

Four-week-old mice were IP injected with diosgenin, blank SLNPs, and diosgenin incorporated SLNP dissolved in saline (5 mg/kg). After 30 min of injection, mice were anesthetized with ketamine + xylazine (87.5 + 12.5 mg/kg) cocktail. Blood was collected from the heart (cardiac puncture) and saved in tubes until extraction. Mice were then sacrificed, and organs were removed, including the brain, heart, liver, spleen, and kidneys. Organs were washed with saline, dried with filter paper, weighed, and kept in ice for extraction.

### Quantification of Diosgenin in Blood and Organs

Blood was pooled into 2-ml microtubes and centrifuged for 2 min, from which serum layer was collected, and methanol was added at a ratio of 2:1 (methanol to serum). Samples were vortexed for 20 s and kept for 1 h at room temperature, which was then centrifuged for 12 min at 16,000 × *g*. The supernatant was separated for extracted drug analysis (Shadkchan et al., 2003).

Folch’s method was followed for drug extraction from organs, with minor modifications (Folch et al., 1957). The tissues were homogenized with a mixture of chloroform/methanol (2:1) to a final volume of 20 ml. The mixture was agitated in a cell disruptor, and the homogenate was centrifuged at 1,000 rpm for 5 min to get the liquid phase. The liquid phase was washed with 0.9% NaCl solution with thorough vortexing for 10 s and centrifuged at 2,000 rpm for 1 min to get two separate phases. The lower chloroform phase was collected for extracted drug analysis.

The concentration of diosgenin was evaluated through UV spectrophotometry at 410 nm of wavelength (Li et al., 2012). The analytes were prepared by mixing the separated phase in 1 ml of acetonitrile. Plasma solution and acetonitrile were blanked before taking reading for analytes. Absorbance was recorded in triplicates. Concentrations of diosgenin in all samples were calculated by interposing with the calibration curve of standard diosgenin and using the slope-intercept formula on the basis of Beer’s law.

### Evaluation of Toxicity

To check the toxicity level, a blood complete profile test was done. Nine 6-week-old mice were IP injected with 100 μL of diosgenin, blank SLNPs, and diosgenin incorporated SLNP (3 mg/ml). Blood was collected after 2 weeks of injections through a cardiac puncture, collected in EDTA tubes, and sent for blood testing. Total leukocyte, lymphocyte, and erythrocyte count was performed (Kim et al., 2014).

### Statistical Analysis

Statistical analysis was done by using GraphPad Prism 5.01 software. The data were analyzed by one-way ANOVA followed by Bonferroni test. *P*-values < 0.05 were considered significant. Data were presented as mean ± SEM with up to two significant figures at a 95% CI.

## Results

### Physio-Chemical Characterization of Stearic Acid Solid Lipid Nanoparticles

#### Scanning Electron Microscopy

**FIGURE 3:**
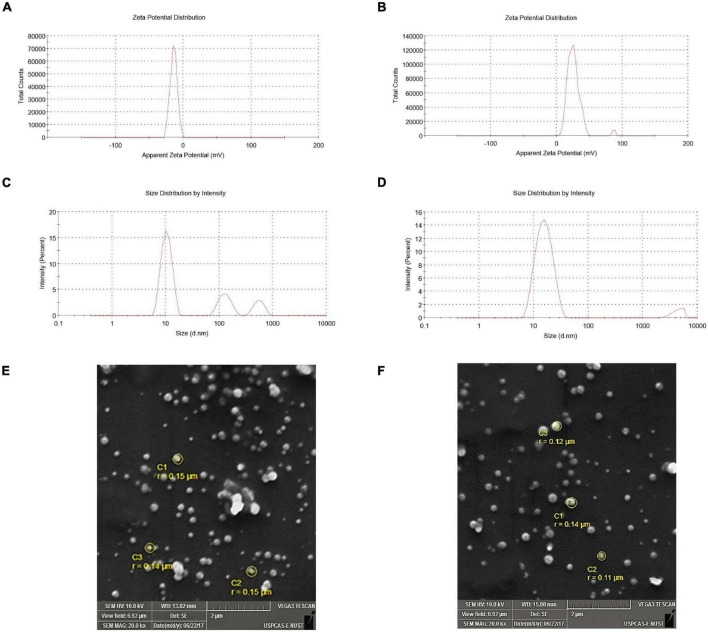
Zeta potential and zeta size of solid lipid nanoparticles. **(A)** Zeta potential of blank solid lipid nanoparticle (-14.1 mV) and **(B)** zeta potential of diosgenin incorporated solid lipid nanoparticles (-26.0 mV) depicting stability. **(C)** Zeta size of blank solid lipid nanoparticles (436.5 nm) and **(D)** zeta size of diosgenin incorporated solid lipid nanoparticles show average size range. SEM images of blank and diosgenin loaded solid lipid nanoparticles were taken at × 20,000 magnifications with size ranging up to 200 nm. **(E)** Blank solid lipid nanoparticles. **(F)** Diosgenin incorporated solid lipid nanoparticles.

SEM images of SLNP are shown in Figure 3, illustrating the morphology and size of SLNP. Morphologically, both blank SLNP and diosgenin incorporated SLNP can be observed from images to be spherical in shape. The size of SLNPs indicated the presence of particles that ranged from a few nanometers to micrometers; however, a significant number of particles were found to be in the range from 20 to 200 nm.

#### Particle Size and Zeta Potential Measurement

Evaluating the zeta potential is an essential indicator for the prediction of stability. The blank SLNPs had a zeta potential (ZP) of -14.1 mV, suggesting low stability with a single peak. Following diosgenin loading, the zeta potential decreased to -26.0 mV as shown in Figure 3, indicating high stability, and the presence of a single peak suggests an increase in monodispersity.

Zeta size graphs show that the formulation with which SLNPs were prepared had particles of different sizes. The pattern showed different peaks, and the average size of blank and diosgenin SLNPs were 436.5 and 15.92 nm.

#### Fourier-Transform Infrared Spectroscopy

**FIGURE 4:**
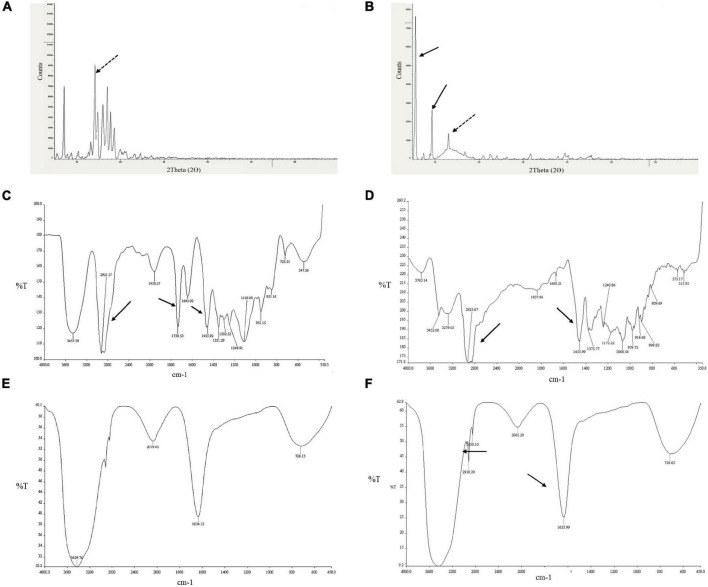
Depiction of diosgenin functional groups with XRD and FTIR patterns. **(A)** XRD peaks for diosgenin. **(B)** Shows peaks for diosgenin that is incorporated in solid lipid nanoparticles. Dotted black arrow indicates diosgenin peak; solid black arrow, solid lipid nanoparticles. FTIR spectrum showing **(C)** Tween 80 peaks, **(D)** diosgenin peaks, **(E)** blank solid lipid nanoparticle peaks, and **(F)** Tween 80 coated diosgenin solid lipid nanoparticle peaks. Arrows indicate characteristic peaks.

The FTIR spectra of diosgenin, Tween 80, blank SLNP, and diosgenin incorporated SLNP, as shown in Figure 4, were performed to confirm the encapsulation of diosgenin in the SLNP. The spectrum analysis showed the presence of broad peak at 3,429 cm^–^, which indicated the presence of hydroxyl group (–OH) stretching, and the peak at 1,633 cm^–^ indicated (C = C) group presence. The intensification of such peaks showed signs of diosgenin encapsulation. The preservation of characteristic absorption bands of Tween 80 in the diosgenin-loaded formulation interprets no significant interruption by the drug on functional groups of Tween 80 coated SLNP. However, the spectrum showed a peak at 2,918 and 2,850 cm^–^, indicating alkane C–H bond stretch, and suggesting H bond interaction between diosgenin and SLNP components. In the spectrum of Tween 80 coated diosgenin SLNP, the specific peaks of both drug and Tween 80 were indicated at a similar position as alone in the spectrum.

#### X-Ray Diffraction Analysis

XRD was performed to identify the physical state of diosgenin, which was incorporated in SLNP, and different patterns of diosgenin, blank SLNP, and diosgenin incorporated SLNP are shown in Figure 4. The data were collected at room temperature in the 2θ range 5.0–69.99°. In this XRD pattern, diosgenin showed characteristic peaks at different ranges, and this was attributed to its crystalline structure in pure drug form. Patterns of blank SLNP and diosgenin incorporated SLNP were quite different from each other but showed the presence of diosgenin characteristic peaks in diosgenin incorporated SLNP. This predicted that SLNP with diosgenin incorporated in its core gave a characteristic peak range of drug, drug SLNP, and blank SLNP that is a bit different with respect to height and range position (Okawara et al., 2013; Manrique-Moreno et al., 2014).

### Diosgenin Release and Entrapment Efficiency of Stearic Acid Solid Lipid Nanoparticle

SLNP capability to entrap and release the diosgenin was evaluated using HPLC.

**FIGURE 5:**
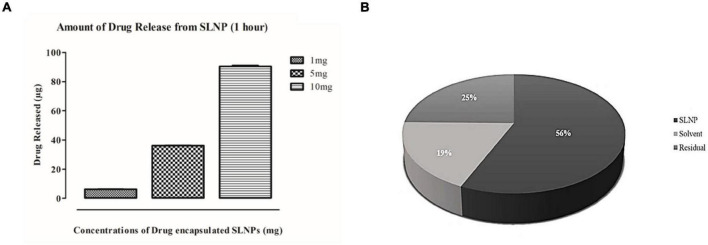
Drug release and entrapment efficiency has been analyzed with HPLC. **(A)** Histogram representing diosgenin release from solid lipid nanoparticles after an hour. Bars show concentration-dependent diosgenin release. **(B)** Pie chart representing percent concentrations of diosgenin present/entrapped in solid lipid nanoparticles, formulation solvent (unentrapped) and residue in syringe filter showing diosgenin is entrapped in solid lipid nanoparticles at higher concentration (56%) then in residue (25%) and solvent have the least concentration of diosgenin (19%).

Results indicate that 56% of the drug was encapsulated in SLNPs while 19% was unentrapped in the solvent and 25% was in residues. These results indicate that the maximum drug got encapsulated in SLNPs (Figure 5A). Furthermore, the efficacy of SLNPs to release the drug was also evaluated, and it was found that diosgenin was efficiently released in the environment (solvent) after 1 h in a dose-dependent manner (Figure 5B).

### Cytotoxic Assay Analysis

The cytotoxic potential of diosgenin and diosgenin incorporated SLNP were evaluated on U87 cells. Both showed U87 cell growth inhibition in a dose-dependent manner. Statistical data analysis (linear regression formula) predicted the IC_50_ value of diosgenin with concentrations (serial dilutions) ranging from 0.001 to 1,000 μM on U87 cells to be 194.4 μM.

**FIGURE 6:**
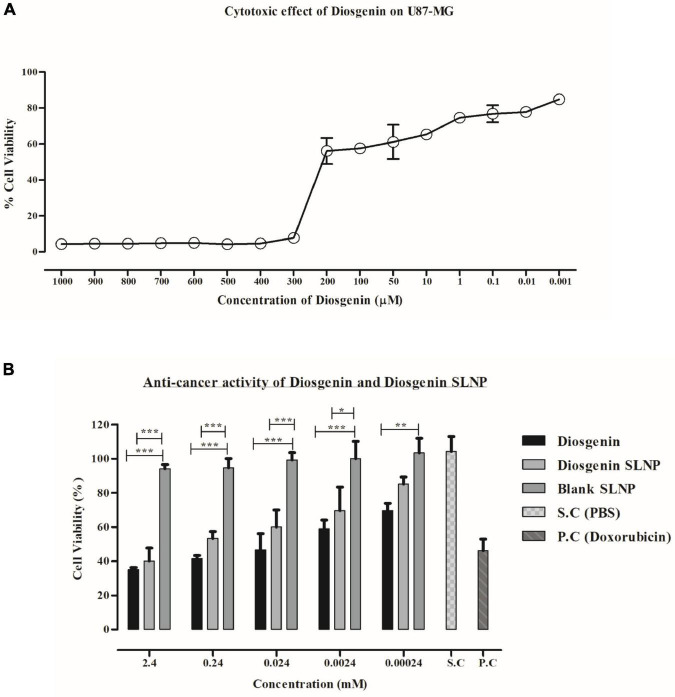
MTT assay. **(A)** U87 MG cell viability at different concentrations of diosgenin depicting dose-dependent cytotoxic effect and IC_50_ value of 18.34 μM. **(B)** Effect of diosgenin and diosgenin incorporated solid lipid nanoparticles on U87 cell viability after 24 h showing both to be toxic on cells with *p*-value less than 0.001 compared with that of blank solid lipid nanoparticles. Results have been presented as mean ± SEM. *p < 0.05, **p < 0.01, ***p < 0.001.

Relative *in vitro* cytotoxic effect of diosgenin incorporated SLNP as compared with the diosgenin and blank SLNP alone was evaluated by MTT assay. Saline was used as solvent control, and doxorubicin was used as a positive control. As depicted in Figure 6, SLNPs were non-cytotoxic to cells comparable with the saline (control). Diosgenin incorporated SLNPs were significantly less toxic to the cells as compared with the diosgenin alone for all tested concentrations (2.4–0.00024 mM). Diosgenin has shown a significant (*p* < 0.001 and *p* < 0.01) anticancer potential against the U87 cancer cells with an IC_50_ value of 0.568 μM as compared with the blank SLNPs, positive control, and solvent control. Our results (Figure 6) thus indicate that SLNP encapsulated diosgenin (IC_50_ value 1.488 μM) is better to be used as an anticancer drug compared with naked diosgenin.

### Analysis of Diosgenin Treatment in Mice for Induced Sickness Behavior

#### Diosgenin Treatment Preserved Locomotory Exploration

**FIGURE 7:**
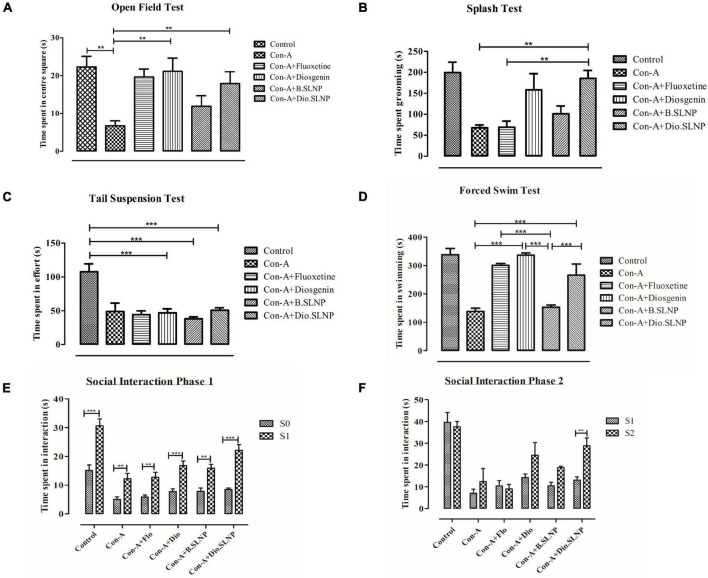
Quantification and comparison of different treatment groups of mice. Control group was subjected to intraperitoneal injection of saline (10 ml/kg body weight), and all other groups were subjected to intraperitoneal injection of Con-A (10 mg/kg prepared) and 30 min later were treated with intraperitoneal injection of fluoxetine (20 mg/kg body weight), diosgenin (100 mg/kg in body weight), blank solid lipid nanoparticles, and diosgenin solid lipid nanoparticles. **(A)** Graph of open field test showing comparison of total exploratory time (seconds) spent in the center square of box after 24 h of exposure with Con-A. Con-A–treated group showed decreased locomotory exploration when compared with other groups. Significant increase in locomotory behavior was observed in Con-A+Diosgenin (*p* < 0.01) and Con-A+Diosgenin solid lipid nanoparticles (*p* < 0.01). **(B)** Sucrose splash test. A significant difference was observed between Con-A vs. Con-A+Diosgenin solid lipid nanoparticles (*p* < 0.01) and Con-A+Flo vs. Con-A+Diosgenin solid lipid nanoparticles (*p* < 0.01). **(C)** Tail suspension test. Con-A group showed decreased mobility as compared with control group, which was not significantly increased in other groups. **(D)** Forced swim test. Con-A group exhibited decreased mobility while significant increase in mobility time is observed in other groups treated with fluoxetine, diosgenin, and diosgenin SLNP; Con-A vs. Con-A+Diosgenin (*p* < 0.001) and Con-A vs. Con-A+Diosgenin solid lipid nanoparticles (*p* < 0.001). **(E,F)** Social interaction test showing phase 1 and phase 2, depicting social interaction and preference respectively. Phase 1 shows measurement of social preference between empty cage (S0) and caged mice (S1) by treated mice in which every group showed significant difference to that of Con-A group, and phase 2 shows social novelty between known caged mice (S1) and new caged mice (S2) by different groups of treated mice in which significant difference is observed between Con-A vs. Con-A+Diosgenin solid lipid nanoparticles (*p* < 0.01). Error bars represent mean ± SEM for one-way and two-way ANOVA followed by Bonferroni’s multiple comparison analysis. Significant values: **p < 0.01, ***p < 0.001.

Exploratory behavior was assessed on the basis of time spent in the arena within set testing time (5 min); more time spent by the subject in the arena indicates a low level of despair. Our results indicate that the saline-treated control group spent maximum time showing exploratory behavior as compared with the Con-A group (control: 178 s vs. Con-A: 54 s, *p* < 0.01) in the center, as shown in Figure 7A. Con-A–treated group showed significantly low exploratory behavior and displayed a significant difference to diosgenin and diosgenin SLNP-treated subjects. Subjects treated with a standard antidepressant, fluoxetine (Con-A+Flo: 157 s; *p* < 0.05), and blank SLNP (Con-A+B.SLNP: 95 s) showed increased exploratory behavior, but there was no significant difference with the Con-A group. Subjects treated with diosgenin only and diosgenin SLNP showed high exploratory behavior and spent more time in the center arena as compared with Con-A and Con-A+B.SLNP groups (Con-A+Dio: 169 s, *p* < 0.01; Con-A+Dio.SLNP: 143 s, *p* < 0.01).

#### Diosgenin Treatment Alleviated Motivational Behavior

Splash test was used to illustrate the self-care behavior of mice when sprayed with sugar solution on their coat. The time spent by the mouse in cleaning its coat and paws was recorded within testing time (5 min). The histogram in Figure 7B elucidates that the control group injected with saline solution spent more time in cleaning or licking itself than other groups. Subjects treated with Con-A spent significantly less time in self-care when compared with saline-treated control group subjects (67.7 s vs. 199.5 s, *p* < 0.01). Treatment with Dio.SLNP significantly improved the grooming of subjects when compared with Con-A and Con-A+Flo groups (Con-A+Dio.SLNP: 185.7 s; *p* < 0.01). Although fluoxetine treatment does not show any improvement in grooming behavior, instead, it is equivalent to the Con-A group with no significance (Con-A+Flo: 68.7 s), treatment with diosgenin only showed marked improvement in grooming behavior (Con-A+Dio: 158.3 s) but shows no significance. However, when treatment with blank SLNP is compared with fluoxetine, it showed an increase in self-care behavior but with no marked significance (Con-A+B.SLNP: 101.2 s). The effect of diosgenin SLNP on self-care behavior was significantly higher than that of fluoxetine-treated subjects (*p* < 0.01), which showed its therapeutic antidepressant effect.

#### Diosgenin Treatment Counteracted Immobility Behavior

In the tail suspension test, the parameter that was focused on was the forceful twisting with rigorous movements to escape the position, which showed the effort of escaping from the position. Among all groups, control mice were active with significantly higher mobility time than all groups (control: 107.3 s; *p* < 0.001). The histogram in Figure 7C elucidates that next to control, Con-A mice were actively putting effort in escape with second-highest time spent in the twisting (Con-A: 65.3 s) with no significant difference. An evident significant difference of saline-treated control group was seen with diosgenin only, blank SLNP, and diosgenin SLNP groups (46.7 s; 38.1 s; 50.7 s; *p* < 0.001). This experiment, however, showed decreased effort put in by most of the treated group as compared with the control and Con-A group, which showed the negative aspect of the test agents rather than being therapeutic.

#### Diosgenin Treatment Assessed Behavioral Despair

In forced swimming, behavioral despair was evaluated by the time spent in mobility or forceful limb movements in the water. Saline-injected subjects showed high mobility with no signs of despair (control: 338.3 s). While the subjects that showed the least time spent was injected with concanavalin-A only (Con-A: 138.6 s), subjects treated with both naked and SLNP coated diosgenin showed an increase in mobility with evident significant difference to Con-A group (Con-A+Dio: 336.8 s; Con-A+Dio.SLNP: 226.2 s; *p* < 0.001) as the standard antidepressant drug, fluoxetine (Con-A+Flo: 301 s; *p* < 0.001). Treatment with both fluoxetine and diosgenin showed the highest and equivalent rate of mobility among test groups but with no significant difference to each other. At the same time, there is an evident significant difference between subject mobility of blank SLNP and diosgenin SLNP groups (*p* < 0.001). Overall, the results (Figure 7D) indicate a positive effect of naked and SLNP coated diosgenin in alleviating the behavioral despair.

#### Diosgenin Treatment Maintained the Social Ability and Novelty

Social interaction test was used to determine the social preference and social novelty of mice. Time spent by mice with empty cage S0, as well as cages with S1 and S2 mice, were recorded and evaluated for behavior among different groups. The saline-treated control group showed more interaction than other groups and are the active ones and showed preference to S1 more than S0 empty cage (Figure 7E). Except for the control group, groups like Con-A+Dio and Con-A+Dio.SLNP from other groups showed a higher preference for S1 than S0, which shows social ability. In phase 2 (Figure 7F), the social novelty was assessed in which it was observed that the test mouse showed preference to the new mouse in S2 cage over the S1 mouse. The most significant difference was observed for groups that were treated with diosgenin and diosgenin SLNPs. Overall results indicate that the treatment restored the ability of subjects for social novelty.

#### Diosgenin Mitigated Neural Inflammation and Morphological Alterations

**FIGURE 8:**
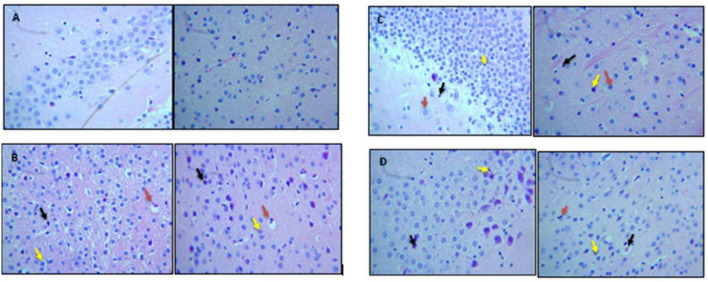
Micrographs showing H&E staining of brain section. This figure represents histological images of **(A)** control, **(B)** Con-A, **(C)** Con-A+Dio, and **(D)** Con-A+Dio.SLNP groups. Red arrow: vacuolization; black arrow: pyknosis; yellow arrow: infiltration of inflammatory cells.

Qualitative analysis of histological slides was performed on the cortical and hippocampus sections of the brain, which were stained with H&E. Micrographs shown in Figures 8A–D represent the brain sections from control, Con-A, Con-A+Dio, Con-A+B.SLNP, and Con-A+Dio.SLNP groups that were taken at 40 × resolutions to observe the presence of pyknosis, neuro-inflammation, and vacuolization in the brain. Histopathological changes in the regions of the brain (cortex and hippocampus regions) for all groups are shown in Figure 8. The control group brain section showed normal glial and neuronal cells in the hippocampal and cortex region. No vacuolization was observed. Con-A–treated mice showed neuro-inflammation with pyknosis, edema, vacuolization, and abundance of inflammatory infiltrates. Interestingly, the brain sections of diosgenin-treated subjects (Con-A + Dio.SLNP) showed a moderate level of inflammatory cells with less pyknosis and gliosis as compared with the Con-A ones. Overall results are indicative of the fact that diosgenin and diosgenin coated SLNPs have been able to decrease the inflammation in the brain region by reduction in inflammatory cells infiltration.

### UV Spectrophotometry of Diosgenin Extracted From Blood and Organs

To access the impact of encasing the diosgenin within SLNPs on the drug load in various organs, the concentration of diosgenin was measured in various organs, including the brain, heart, liver, spleen, and kidneys of the subjects injected with naked diosgenin as well as diosgenin incorporated SLNP.

**FIGURE 9:**
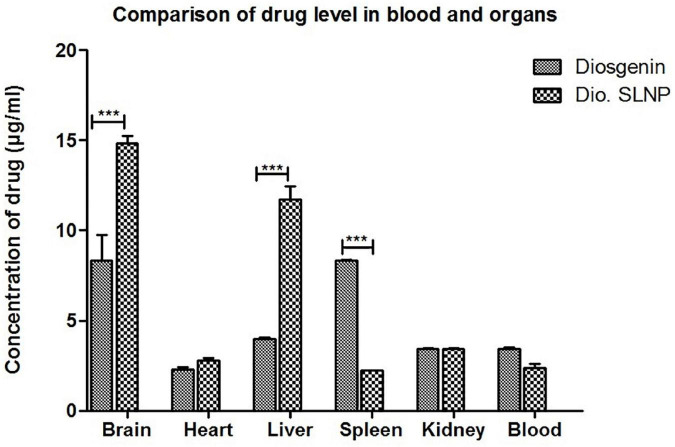
Concentrations of diosgenin level in brain, heart, liver, spleen, kidney, and blood, which was administered intraperitoneally (5 mg/kg in saline 10 ml/kg body weight). Comparison shows a significant difference of diosgenin level in brain, liver, and spleen when administered alone and when encapsulated in solid lipid nanoparticles. ***p < 0.001.

Spectrophotometric analysis revealed that influx of diosgenin was more toward the spleen (Dio: 8.3 μg/ml vs. Dio.SLNP: 2.2 μg/ml; *p* < 0.001) when injected naked; however, when incorporated with SLNPs, there was a significant shift in the influx of diosgenin toward the brain (Dio: 8.35 μg/ml vs. Dio.SLNP: 14.8 μg/ml; *p* < 0.001) and liver (Dio: 3.9 μg/ml vs. Dio.SLNP: 11.7 μg/ml; *p* < 0.001) while levels were reduced in the blood (Dio: 3.4 μg/ml vs. Dio.SLNP: 2.3 μg/ml) and spleen (Figure 9). In addition, there was a significant increase of diosgenin concentrations in the brain, indicating a target-specific delivery of diosgenin toward the brain as desired (Dio: 8.3 μg/ml vs. Dio.SLNP: 14.8 μg/ml; *p* < 0.001). There is, however, a need to reduce the influx toward the liver to make the delivery more target specific. Heart and kidneys had almost equal levels of diosgenin for both groups with no significant difference.

### Toxicity Evaluation From Blood

Complete blood profile (CBP) was done for mice of all four mice groups to elucidate the induced toxicity (if any) after injecting the formulations. There was no significant difference in neutrophil erythrocyte and leukocyte count among all test groups (Table 2), indicating that no toxicity was induced after treatment with diosgenin and SLNPs encapsulating diosgenin. There was a decrease in the leukocyte (Table 2) count in the diosgenin-treated mice group but did not show any significant difference with other groups.

**TABLE 2:** Blood complete profile of different mice group shows level of different cell count (leukocyte count, neutrophil count, and erythrocyte count) after treatment with diosgenin, diosgenin SLNP, and blank SLNP for toxicity analysis.

Parameters (preferred range)	Diosgenin	Diosgenin SLNP	Blank SLNP	*P*-value
*Leukocyte* *(4.5–10 × 10^9^/L)*	2.9	49.1	7.0	Not significant
	2.2	70.6	7.6	Not significant
	3.2	77.8	9.4	Not significant
*Neutrophil* *(60–80%)*	5.9	92.6	8.4	Not significant
	6.9	81.4	8.6	Not significant
	5.7	80.5	8.3	Not significant
*Erythrocyte* *(5.1–7.5 × 10^12^/L)*	4.1	78	4.2	Not significant
	4.8	80	9.6	Not significant
	8.9	69	6.8	Not significant

## Discussion

MDD is difficult to treat with morbidity, especially in comorbidities originating inside the brain. Depression comorbidity worsens treatment outcomes, while antidepressant treatment may interfere with the efficacy of cancer treatment regimes. Crossing BBB is an additional limiting factor for many molecules. An ideal molecule, in this scenario, will be one that can cross the BBB easily and can treat cancer and depression at the same time. Based on the existing literature, diosgenin merited an inquiry for anticancer and antidepressant efficacy as well as its ability to cross the BBB riding a solid nano-vehicle. The current study aimed to evaluate dual therapeutic and preventive potential (anticancer and antidepressant) of diosgenin and diosgenin encapsulated SLNPs against GBM cell line U87-MG and Con-A–induced sickness behavior syndrome, respectively.

SLNPs carrying diosgenin were prepared, and their anticancer and antidepressant potential was accessed *in vitro* using the U-87 cell lines and *in vivo* using a rodent model of depression (Nazir et al., 2021). Our results present a fascinating insight into the potential of diosgenin as a desired dual-action molecule: anticancer and antidepressant activity. Diosgenin encapsulated SLNPs were hemocompatible and effectively permeated the BBB to deliver the compound of interest. Moreover, the effectivity of diosgenin and diosgenin encapsulated SLNPS in amelioration of measures of sickness behavior and decrease in cancer cell viability are all but signs of its potential as a pleiotropic drug candidate. Different aspects of the experiments are discussed hereunder.

Diosgenin (3β-hydroxy-5-spirostene), a herbal steroidal sapogenin extracted from *Trigonella foenum-graecum*, *Dioscorea bulbifera*, and *Cheilocostus* sp., has been found to decelerate the development of neuropathic pain in diabetic rats by decreasing oxidative stress and inflammation (Kiasalari et al., 2017). Diosgenin presented promising therapeutic and preventive results when studied against neurodegenerative diseases, diabetes, allergic diseases, cardiovascular disorders, obesity, and cancer (Jesus et al., 2016). Structurally, diosgenin consists of a hydrophilic sugar moiety linked to a hydrophobic sapogenin, showing similarity to steroids and cholesterol, and is poorly soluble in water. To resolve the solubility issue and to enhance transport across the BBB, diosgenin encapsulated SLNPs were developed and characterized in the current study.

Stearic acid–based SLNPs were developed by solvent injection method and coated with Tween 80 to facilitate transport across the BBB; Tween 80 functionalization was confirmed by FTIR. Particle size and shape were assessed through SEM, and the majority of the particles were found to be in the range of 20–200 nm. Particle size determines the circulation time in blood because particle sizes above 200 nm accumulate in the liver and are removed from circulation due to activation of the complement system (Hoshyar et al., 2016). There was a significant increase in biodistribution of diosgenin in the brain after 1 h of diosgenin encapsulated SLNPs as compared with diosgenin alone, which can be due to small particle size and Tween 80 functionalization. It has been speculated that either Tween 80 coating causes adsorption of Apo-E proteins and then are recognized by LDL receptors on epithelial cells and find their way into the epithelial cells where a drug is released, or it helps to open up the tight junctions of BBB and inhibit p-glycoproteins (Hartl et al., 2021). Our results are in accordance with previously published literature where increased delivery of doxorubicin, loperamide, NMDA receptor antagonist MRZ 2/576, and tubocurarine across the BBB via nano-carriers coated with Tween 80 was reported (Zlokovic, 2008; Abbott, 2013). Increased therapeutic efficiency is another beneficial aspect of Tween 80 coated nanoparticles; for example, in the GBM rat model, Tween 80 coated doxorubicin leads to 40% cure (Chacko et al., 2018). The zeta potential of the SLNPs was negatively charged, categorizing them as anionic SLNPs. Negative charge makes nano-carriers susceptible to increased cellular uptake via cationic sites due to the repulsive energy they face from negatively charged sites in the cell membrane (Behzadi et al., 2017; Foroozandeh and Aziz, 2018). The higher negative charge also ensures the stability of nanoparticles, and in the current study, diosgenin encapsulated SLNPs possess -26.0 mV zeta potential possessing stability and low aggregation properties. However, Tween 80 coating also endorses steric stability of nanoparticles (Honary and Zahir, 2013). For toxicity analysis, hemocompatibility testing was performed to evaluate the effect of engineered diosgenin nanoparticles on erythrocyte, leukocyte, and neutrophil counts. Blood is the gateway for nanoparticles, which leads them to the target organ. Critical interactions between nanoparticles and blood components may disrupt the structure and function of these components, causing toxic effects (de la Harpe et al., 2019). In the current study, diosgenin encapsulated SLNPs showed hemocompatibility as erythrocyte, leukocyte, and neutrophil count remained regular after administration of diosgenin encapsulated SLNPs for 2 weeks, which demonstrates the safety of diosgenin and diosgenin nanoparticles (Table 2). After characterization and toxicity analysis, diosgenin encapsulated SLNPs were tested for *in vitro* anticancer and *in vivo* antidepressant potential using GBM U-87 cell line and sickness behavior animal models separately. Diosgenin encapsulated SLNPs showed promising therapeutic potential in both MTT assay (Figure 6) and the concanavalin-A–induced sickness behavior model of depression in mice (Figure 7).

Diosgenin and diosgenin encapsulated nanoparticles show dose-dependent antiproliferative activity against GBM cell line, and there was no significant difference between the cytotoxic potential of diosgenin and diosgenin encapsulated SLNPs. This leads to the notion that encapsulation of diosgenin in the stearic acid and P80 coating does not affect its anticancer potential. IC_50_ of diosgenin was 194.4 μM against the U-87 cell line. The cytotoxic potential of stearic acid–based diosgenin encapsulated nanoparticles against the U-87 cell line has not been reported previously. However, *in vitro* anticancer activity of diosgenin against breast carcinoma, human laryngeal carcinoma, melanoma, osteosarcoma, prostate carcinoma, hepatocellular carcinoma, and human erythroleukemia have been documented due to its pleiotropic effects such as apoptosis, cell cycle arrest, inhibition of angiogenesis, invasion, and metastasis via affecting mediators involved in inflammatory cascades such as NF-kb, STAT3, AKT, and JNK (Sethi et al., 2018; Cai et al., 2020).

**FIGURE 10:**
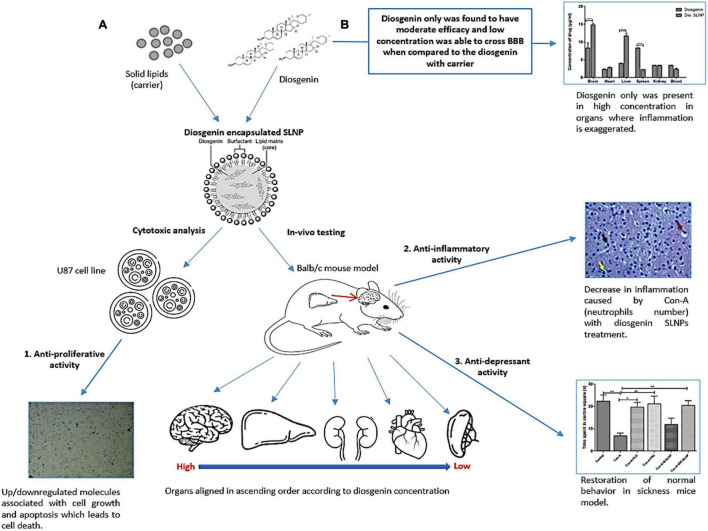
Proposed mechanism of diosgenin encapsulated SLNPs and diosgenin only activity on Con-A–induced sickness in animal model and *in vitro* analysis. **(A)** Con-A induces sickness in animal model due to its ability of triggering immune system, which results in the production of inflammatory cytokines. Diosgenin when in carrier decreases the inflammation and its capability of crossing the BBB boosts up while being least toxic. This formulation also restored the normal behavior in sickness mice model and showed anti-proliferative activity in cell line by the regulation of apoptotic molecules. **(B)** Diosgenin only still shows its therapeutic capability, but being steroid in nature, it is not soluble and its capability to cross the BBB is low. It gets highly concentrated in spleen and liver, which is the site of inflammatory responses.

In the next *in vivo* experiments, the behavioral effect of diosgenin and diosgenin encapsulated SLNPs was analyzed using the Con-A–induced sickness behavior model. Con-A has previously been reported to induce sickness behavior by activation of the immune system leading to neuroinflammation via inflammatory cytokines (Sass et al., 2002). Pro-inflammatory cytokines have already been documented for their pivotal role in the induction of sickness behavior (Dantzer, 2009) and cancer progression (Dinarello, 2006). Sickness behavior in cancer patients can be a result of disease progression or treatment (Santos and Pyter, 2018). It has been associated with poor quality of life and abridged treatment compliance in chronic disorders, including cancer (inflammation, sickness behavior, and depression) (Maes et al., 2012). Pro-inflammatory cytokines are a shared link between sickness behavior and cancer (Myers, 2008). Con-A–induced depressive and anxiety-like behavior in the current study was in accordance with previous reports (Biesmans et al., 2016; Nazir et al., 2021). In the current study (Figure 10), diosgenin and diosgenin encapsulated SLNPs significantly protected the mice against behavioral shift induced by Con-A as indicated by improved social interaction and novelty in social interaction test, optimal grooming activity in splash test, and decreased immobility time in FST. The antidepressant potential of diosgenin in an animal model of depression or sickness behavior has not been explored previously; however, its antidepressant activity in ovariectomized rats has been documented (Ho et al., 2012). Our results are in accordance with these findings and help in extrapolating the potential of nanoparticle-enabled diosgenin delivery across BBB for intracranial inflammatory and cancerous conditions.

The development of a therapeutic agent against brain pathologies has always been a challenge due to the complexity of the disorder and the disability of the therapeutic agent to reach the brain. However, pathologies like cancer and MDD have been even more challenging to treat due to the fact that both support each other in the pathogenesis of the disease (Gramatzki et al., 2020). Due to this reason, many therapeutic drugs have shown less efficacy against such pathologies. That is why scientific research has now been more focused on therapeutic entities, which have dual nature as antidepressant and anticancer. Various studies have shown the potential effect of antidepressants in MDD and in cancers. Preclinical studies have shown the antiproliferative and antitumor activity of certain antidepressants that are prescribed for cancer patients suffering from depression, such as fluoxetine (FLT). Antidepressants like fluoxetine have been used for MDD treatment, but experimental studies have also indicated its cytotoxic effect on glioma cells by inducing apoptosis in the cell lines (Petersen et al., 2013). Studies have also shown a high concentration of FLT in the brain, which indicates its effectiveness in glioma treatment (Song et al., 2015). Previous research studies like these on an antidepressant acting as a pleiotropic agent goes in concordance with our experimental findings for diosgenin showing both antidepressant and anticancer effects. However, studies have shown patients facing drug resistance and less effectiveness of the tested drugs due to acid degradation, low drug absorption, and low to none target specification due to which drugs are eliminated from the body (Alam et al., 2018; Sharifi-Rad et al., 2021). To overcome such drawbacks, scientific research has been done at a greater extent to devise a carrier system that successfully delivers the drug to its specific target for maximum therapeutic effectiveness via systemic circulation. Nanocarrier has been under high considerations for safe and effective drug delivery, high drug bioavailability and stability, and effective drug encapsulation and release efficiency. Recent studies have shown the unique ability of SLNPs for its size range, biocompatibility, and encapsulation of lipophilic drugs that basically keeps the drug safe from enzymatic degradation and increases its half-life in blood (Porter et al., 2007; Li H. et al., 2009; Neupane et al., 2014). The experimental study has shown the ability of curcumin SLNPs that effectively cross the BBB in mice inducing behavioral changes (He et al., 2016). This study supports our experimental data for diosgenin SLNP, which has also shown a high concentration of diosgenin in the brain when injected with diosgenin SLNP as compared with the naked drugs when injected into mice. This supports the statement that P80 coated SLNP can effectively cross the BBB while carrying a drug load (Tian et al., 2011). Liu et al. (2016) have suggested in a study about the carrier’s drug depot effect in which carrier can help in the stable release of hydrophobic therapeutic drugs into the systemic circulation without compromising the drug integrity and maintaining its half-life as well (Wang et al., 2015; Qu et al., 2016). Our study on diosgenin SLNP has also supported this phenomenon that diosgenin being a hydrophobic drug can be stable when encapsulated in SLNP and there is a gradual release of the drug from SLNP, which can be an advantage for cancer therapeutics because of the stable and sustained release of the drug from SLNP that can provide continuous and persistent protection against the pathology for a longer period (Qu et al., 2016). Studies showed that one of the drugs (doxorubicin), which is highly cytotoxic to cells, can be stabilized when encapsulated in SLNP and still be cytotoxic enough on cells but in a controlled manner (Battaglia et al., 2014), supporting our experimental analysis for SLNPs. All of the studies that have been mentioned have supported this study hypothesis for introducing a drug that has dual nature of being antidepressant and anticancer as well and still can show its effectivity in the brain region by crossing the BBB with the help of SLNPs.

Conclusively, diosgenin and diosgenin encapsulated SLNPs show anticancer and antidepressant potential, while the diosgenin encapsulated nanoparticles are hemocompatible and lead to improved drug delivery across the BBB. Furthermore, these findings are based on an *in vitro* and *in vivo* model study, which is not sufficient to evaluate its effectiveness at clinical level for which clinical trials are required. To date, there is lack of knowledge regarding the molecular pathway that diosgenin follows and its exact interaction with cellular components. This requires further investigation, which will shed light on diosgenin’s therapeutic effects in MDD and GBM pathology. Due to the gradual development of such pathologies, there is requirement of treatment throughout the patient’s life for which it is a must to asses patient’s treatment outcomes. This needs a systematic way to design experiments that will assess the effectivity of therapeutic agents and management of symptoms. Further studies using an animal model of GBM and other cancers can explore the underlying mechanism of these pleiotropic effects of stearic acid–based diosgenin encapsulated nanoparticles. Combinatorial therapy is also gaining popularity and including diosgenin with other potential drugs can show synergistic effects in the treatment of GBM that will somehow target the heterogenic and resistant nature of GBM.

## Conclusion

This study demonstrates that the BBB obstacle can be overcome and elucidates the feasibility of penetrating the BBB effectively by formulating efficient carrier nanoparticles that can encapsulate a drug and transport it to the target area. This formulation of SLNPs with diosgenin was used for glioma cell line that proved to be efficient to control tumor cell proliferation. The size, stability, and low toxic nature showed through experimentations have shown its biocompatible nature. Con-A model of sickness was successfully generated and confirmed as reported previously (Nazir et al., 2021). Interestingly, diosgenin coated/incorporated SLNPs (Tween 80 coating) have experimentally shown significant improvement in Con-A–induced sickness behavior as evident from various extensive behavioral tests performed. The reversal of this behavior was through reduction in inflammation as confirmed through histopathology imaging of brain. Promisingly, our results have proven the targeted drug delivery of SLNPs to brain in higher concentrations by rerouting it to brain from other organs specifically spleen. Conclusively, the safety profile of diosgenin along with the efficacy of SLNPs to cross the BBB and deliver the drug on its target adds immense excitement to future experiments on this subject. Prospects of diosgenin must be tested in an *in vivo* model of cancer depicting depressive features in conjunction with different nanoparticles to optimize its efficacy and safety. Natural products for brain pathologies can provide safer alternatives to the current cytotoxic chemotherapeutic regimens against cancer.

## Data Availability Statement

The raw data supporting the conclusions of this article will be made available by the authors, without undue reservation.

## Ethics Statement

The animal study was reviewed and approved by the Internal Review Board (IRB), National University of Sciences and Technology (NUST), Pakistan.

## Author Contributions

AJ, RF, and IK designed the study, validated the experimentations, and critically revised the article. HK performed the experiments, generated the data, and wrote the original article. SN assisted in study design, curated the data, and revised the article. All authors contributed to the article and approved the submitted version.

## Conflict of Interest

The authors declare that the research was conducted in the absence of any commercial or financial relationships that could be construed as a potential conflict of interest.

## Publisher’s Note

All claims expressed in this article are solely those of the authors and do not necessarily represent those of their affiliated organizations, or those of the publisher, the editors and the reviewers. Any product that may be evaluated in this article, or claim that may be made by its manufacturer, is not guaranteed or endorsed by the publisher.
